# Simulation and analysis of hydraulic transition process based on mechanical hydraulics

**DOI:** 10.1371/journal.pone.0295044

**Published:** 2023-12-29

**Authors:** Chaoyu Chen, Qingbiao Zhan

**Affiliations:** 1 Department of Intelligent Manufacturing and Information Engineering, Fangchenggang Vocational and Technical College, Fangchenggang, China; 2 Guangxi Chongzuo Xianggui Sugar Industry Co., Ltd, Chongzuo, China; KTH Royal Institute of Technology: Kungliga Tekniska Hogskolan, SWEDEN

## Abstract

With the scarcity of water resources in various regions, the pressure on water supply projects is also increasing, which has led to a sharp increase in the water hammer effect in water supply pump projects. In response to this issue, the study proposes to apply a unidirectional pressure regulating tower based on mechanical hydraulic technology to water hammer protection in water supply pumps. In addition, the study also optimizes the calculation method of water hammer and designs one simulation software, which is used to simulate and analyze the proposed water hammer protection measures. The study first determines through simulation software that the optimal initial water level of the unidirectional pressure regulating tower is 2m and the optimal diameter of the make-up water pipe is 600 mm. Afterwards, simulation analysis is conducted on different water hammer protection measures, and it is found that the maximum pressure of the water hammer under the proposed protection measures is the lowest, at 14.8m, which was lower than the comparison measures. In addition, according to expert ratings, the average protective effect rating of protective measure 1 by relevant technical personnel is 9.4 points, which is better than the comparative measure. The above results indicate that through the simulation analysis of hydraulic transition process based on mechanical hydraulics, it can be found that the water hammer protection measures proposed in the study have good protective functions and can effectively reduce the water hammer effect in water supply engineering.

## 1. Introduction

With the rapid development of industrialization and automation, mechanical hydraulic (MH) technology, as an important power transmission technology, has been widely used in the engineering field [1]. MH has the advantages of high efficiency, stability and reliability, and plays an important role in the simulation analysis and research of hydraulic transition process (HTP) [2]. HTP refers to the process in which a liquid changes from a static state to a stable flow state in a pipeline or device, involving changes in parameters such as velocity, pressure, and flow of the liquid [3]. Therefore, the simulation analysis and research of HTP can deeply understand the working mechanism of the hydraulic system and optimize the performance of the system [4, 5]. However, at present, there are still some problems in the simulation analysis of HTP of MHs [6, 7]. For example, there is a lack of experimental verification for practical engineering problems, and the existing simulation tools and methods have certain limitations in dealing with multi-parameter changes [8, 9]. Therefore, this study aims to conduct simulation analysis and research based on the HTP of MHs. Through modeling and simulation of the changes of liquid velocity, pressure, flow and other parameters, the working mechanism of the hydraulic system in the HTP is deeply understood and methods to optimize the performance of the system are explored. The novelty of this research is mainly reflected in the following aspects: First, the HTP based on MH pressure is taken as the research object, and the dynamic characteristics and parameter changes of the hydraulic system in the transition process are fully considered. Secondly, the method of simulation modeling and experimental verification is used to study from both theoretical and practical levels to improve the ability of simulation and analysis of HTP of hydraulic system. Then, the actual engineering problems will be studied to improve the stability and efficiency of the hydraulic system by optimizing the performance of the system. To sum up, the HTP simulation analysis in this study is of great significance and innovation in the field of MHs, which can provide theoretical guidance and engineering practice support for the design, optimization and troubleshooting of hydraulic systems. This paper is mainly divided into five parts. The first part is related research and analysis of MH technology and HTP. The second part is the construction and optimization of water hammer calculation method of water supply pumping station. The third part is to model the unidirectional pressure regulator based on MH technology and explain its water hammer protection mechanism. The fourth part is to build the simulation system and analyze the simulation results. The fifth part is the conclusion. The abbreviations in this paper are shown in Table 1.

**Table 1:** Meets the table.

Abbreviation	Full name
WHE	Water hammer effect
HTP	Hydraulic transition process
MH	Mechanical hydraulic
UPRT	Unidirectional pressure regulating tower

## 2. Literature review

As the rapid growth of hydraulic technology, its application fields are becoming increasingly widespread. Hong et al. established a numerical analysis method to calculate the equilibrium and internal wall water pressure of the caisson during hydraulic surge. They conducted a surge experiment using a caisson sample in a water tank to verify the method of two boxes. Through engineering examples, they provided the water pressure on the inner wall of the caisson in equilibrium and sudden sinking states. The research outcomes indicated that this method was effective in calculating the water pressure of caissons during water surges [10]. Chen’s team studied the influence of compensation chamber parameters on sprinkler performance through response surface methodology. They established an orthogonal decomposition model that took into account the height and diameter of the compensation chamber, with the aim of achieving optimal water spray performance. Their research had a nozzle design method that provided a reference for optimizing water sprinklers. The experimental findings denoted that the minimum compensation pressure of the nozzle was directly proportional to the height of the compensation chamber and inversely proportional to the diameter of the compensation chamber [11]. Jose et al. used a compensator to counteract the vibration of the brake and partially isolate the transient pressure signal. They used an excavator model for experimental verification of the method. They conducted experiments under different conditions while using a conventional system as a control. For the safety of the experiment, they analyzed its stability and ensured the stability of the system through their developed controller. The research findings expressed that their cost functions reduced vibration amplitude by 58% and 41%, respectively [12]. Chen’s team proposed a second level evolutionary algorithm to improve multi-objective, and conducted multi-objective optimization on the basis of second level evolution. Through consideration of multi-objective function constraints, including restrictions on various parameters such as hydraulic oscillation, they used the proposed optimization scheme to conduct simulation tests on actual hydraulic turbine units under load rejection conditions. The experimental results denoted that their second level evolutionary algorithm had excellent comprehensive performance, and even high-temperature gas reactor could operate stably [13].

With the increasing application range of hydraulic transition, more methods are being applied to the HTP. Dejam derived the dispersion coefficient of the transition of crack shape to advection dominant flow, and provided an analytical expression for the diffusion of tracer in hydraulic fractures. Different hydraulic fracture models were established, which had three shapes: rectangular, triangular, and elliptical, to determine the influence of crack shape on the dispersion rate of tracer. The experimental results indicated that the dispersion rate of the tracer with porous walls was smaller than that with non-porous walls [14]. Iben et al. proposed a three-dimensional to one-dimensional coupling of hydraulic flow, in which hydraulic robustness was strong and human effects at the coupling interface do not occur. Due to the coupling variables present in local virtual units, spatial transformation was simulated through the discontinuity of abnormal components of momentum. They established a barotropic equation of state under the condition of industrial simulation of high pressure, taking into account the cavitation phenomenon. The outcomes indicated that this was suitable for moderate fluctuations [15]. Zhou’s team calculated fluid dynamics and discrete element method, and studied the hydraulic transport value of coarse particles by combining them. On this basis, they predicted the relationship between increasing the conveying speed and pressure characteristics when the volume of the feed solid remained constant. They analyzed the numerical results in flow and force, and proposed a phase diagram to identify the flow particle model and its transformation. They established a relationship for predicting pressure drop based on the data. They compared experimental data on the radial distribution of solid particles and concluded that the model was effective [16]. Xu et al. set up a water tank in the laboratory to study the impact of water jumping on the movement of individual particles and sludge. The process of particle motion was recorded using a high frame rate camera, and the relationship between sediment and hydraulic jump was then statistically analyzed. Due to the resistance attached to the hydraulic jump, the particles in the upstream would gradually slow down and eventually remained stationary during the hydraulic jump. The research results denoted that particle size affected particle motion [17].

The above research not only indicated that MH technology has been widely applied in multiple fields, but also indicated that there were various methods applied to HTP. However, there is still a lack of research on the combination of MH technology and HTP. To fill the research gap in this section and promote the development of the HTP field, the research will apply a UPRT based on MH technology to the HTP of the water supply pump station. It is hoped that through simulation analysis of this process, reasonable suggestions will be provided for the safe operation of the water supply pump station.

## 3. Construction of calculation method for water hammer when water supply pump station stops pumping

The water supply pump station is one of the important facilities for urban water supply, and its main function is to transport the water source required by urban residents to various parts of the city. However, during the water supply, water hammer phenomena may occur due to various reasons, leading to consequences such as pipeline rupture and pump damage, seriously affecting the quality of life of urban residents. Therefore, it is of great significance to study the phenomenon and impact of water hammer when the water supply pump station stops pumping. This section mainly constructs the calculation method of water hammer for the water supply pump station, to facilitate the subsequent simulation of HTP.

### 3.1. Construction of water hammer calculation method for water supply pumping station

**Fig 1:**
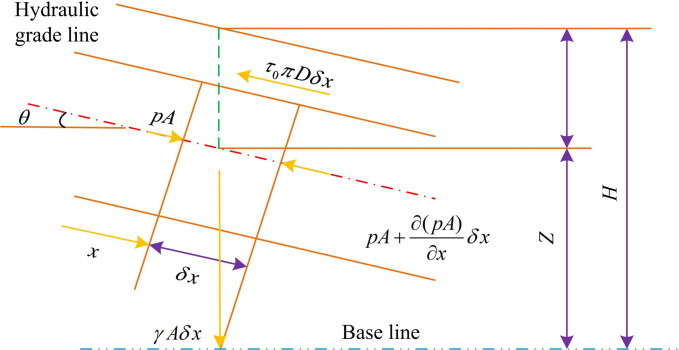
Control volume of equations of motion.

In the water supply pump station, the basic equation of water hammer consists of equations of motion and continuity, of which the equations of motion is the momentum change of water under pressure. The control volume of equations of motion is shown in Fig 1.

As shown in Fig 1, *x* means the coordinate distance from the starting point of the control body on the pipe axis; *θ* denotes the angle between the pipeline and the horizontal line. Through force analysis, the surface positive pressure *pA* and $pA+\frac{\partial (pA)}{\partial x}\delta X$, the shear stress *τ*_0_ on the side of the prism, and the gravity component *γAδX* sin *θ* are the forces acting on the cross-section. According to the momentum theorem, the equation for calculating the total force acting on the control body is shown in Eq (1).


$$\rho A\frac{dv}{dt}\delta X=pA-\left[pA+\frac{\partial (pA)}{\partial x}\delta X\right]-\tau_{0}\pi D\delta X+\gamma A\delta X\sin\theta$$ (1)

**Fig 2:**
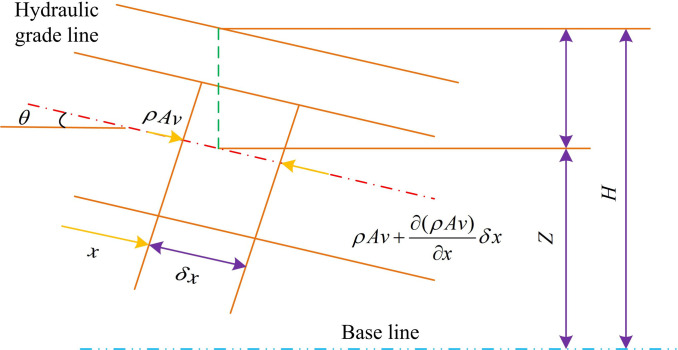
Continuous equation pipeline control body.

The pipeline control body refers to an object that controls fluid flow under continuous equations, which can be a pipeline, container, water turbine, etc. Among these objects, the control body is a fundamental component of the water hammer of the water supply pump, which receives the energy of the fluid and transfers it to the pipe wall to achieve control of the fluid. The continuous equation pipeline control body is shown in Fig 2.

In Fig 2, there is a conservation of mass theorem, which states that the difference in water quality between the inflow and outflow of the control body is equal to the change in water quality over time within the control body. The expression is shown in Eq (2).


$$\rho Av-\left[\rho A+\frac{\partial (\rho Av)}{\partial x}\delta X\right]=\frac{\partial (\rho A\delta x)}{\partial t}$$ (2)

The basic equation of water hammer is a pair of partial differential equations that solve the head and flow velocity of the pressure measuring pipe. There is generally one of the most commonly used methods for numerical solution of quasilinear hyperbolic partial differential equation, one is the method of characteristics, the other is the difference method. The finite difference method is one of the numerical methods for solving differential equations. In the finite difference method, the derivative of the control equation is replaced by the difference quotient, and the hydraulic parameters of the fluid are determined by solving the function values on the grid points. The finite difference method is divided into explicit difference method and implicit difference method. The partial derivative term of the explicit difference method is expanded on the time layer of the known variable, and there is only one unknown node in the grid node, and the equation system itself can solve the unknown node. The explicit difference method is more suitable for calculating rapidly changing water flow phenomena due to its limited time step due to stability conditions. Therefore, the explicit difference method is commonly used in the calculation of water hammer in water pumps. In the explicit difference method, the partial differential equations of head H and flow Q are shown in Eq (3), respectively.


$$\left\{\begin{array}{c} \frac{\partial H}{\partial t}=\frac{H_{i}^{j+1}-0.5(H_{i}^{j+1}+H_{i+1}^{j})}{\Delta t}\\ \frac{\partial H}{\partial x}=\frac{H_{i+1}^{j}-H_{i-1}^{j}}{2\Delta x}\\ \frac{\partial Q}{\partial t}=\frac{Q_{i}^{j+1}-0.5(Q_{i}^{j+1}+Q_{i+1}^{j})}{\Delta t}\\ \frac{\partial Q}{\partial x}=\frac{Q_{i+1}^{j}-Q_{i-1}^{j}}{2\Delta x} \end{array}\right.$$ (3)

After substituting Eq (3) into the water hammer equation, the difference equation shown in Eq (4) can be obtained.


$$\left\{\begin{array}{c} Q_{i}^{j+1}=\frac{1}{2}(Q_{i-1}^{j}+Q_{i+1}^{j})-\frac{gA}{2}(\frac{\Delta t}{\Delta x})(H_{i+1}^{j}-H_{i-1}^{j})-\frac{f}{2DA}\Delta t\frac{1}{2}(Q_{i-1}^{j}+Q_{i+1}^{j})|Q_{i}^{j}|\\ H_{i}^{j+1}=\frac{1}{2}(H_{i-1}^{j}+H_{i+1}^{j})\frac{1}{2}\frac{\Delta t}{\Delta x}\frac{a^{2}}{gA}(Q_{i+1}^{j}Q_{i-1}^{j}) \end{array}\right.$$ (4)

**Fig 3:**
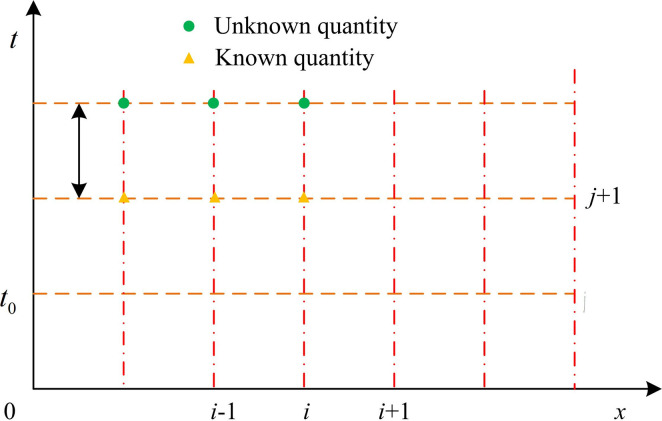
Schematic diagram of the difference grid of the explicit difference method.

From Eq (4), the equation only includes known quantities at the beginning of the time period. Therefore, the head *H* and flow *Q* at the end of the i-th section time period can be obtained through Eq (4). The difference grid diagram of the explicit difference method is shown in Fig 3.

The grid sensitivity of differential mesh refers to how the change of grid step size affects the stability and accuracy of the calculation results when the explicit difference method is used for numerical calculation. In the explicit difference method, the value of the next time step needs to be calculated using the value of the current time step and the value of the adjacent grid points. The size of grid step determines the distance between adjacent grid points, so the change of grid step size will directly affect the accuracy and stability of numerical calculation. When the grid step size is large, the distance between adjacent grid points is far, which may reduce the precision of numerical calculation. In this case, the numerical calculation may produce a large error, resulting in the result deviating far from the real solution. When the grid step size is small, the distance between adjacent grid points is closer, and the precision of numerical calculation is higher. However, a smaller grid step size also means that more computational steps are required, which increases the time and resource consumption of the computation. Therefore, the grid sensitivity of differential grids requires a trade-off between accuracy and computational efficiency. In order to obtain better water hammer calculation results, different grid sensitivity values were obtained according to the actual situation of different points in the water supply pump station, so a specific sensitivity value could not be provided.

### 3.2 Water hammer calculation algorithm based on method of characteristics

The characteristics differential grid algorithm uses characteristics as boundary conditions for difference calculations, which can better reflect the characteristics of the flow field. Compared with traditional difference grid algorithms, the characteristics difference grid algorithm has the following characteristics: High accuracy: The characteristics difference grid algorithm adopts a high-precision difference format, which can simulate small changes in the flow field well. Strong adaptability: The characteristics difference grid algorithm can be applied to various types of flow field problems, including homogeneous and heterogeneous flows. High efficiency: The characteristics difference grid algorithm can quickly solve all boundary conditions and achieve fast convergence in calculations. Flexibility: The characteristics difference grid algorithm can flexibly adjust parameters such as mesh size, shape, and position according to actual needs to adapt to different flow field problems. In general, the characteristic difference grid algorithm is an efficient and flexible flow field calculation tool, which can better reflect the characteristics of the flow field and provide better simulation support for fluid dynamics calculation. By using the method of characteristics, the partial differential equations are transformed into ordinary differential equation, and then the equations are solved. In using the method of characteristics to calculate the water hammer, it is first necessary to substitute the equation of flow rate and velocity into Eqs (1) and (2) for variation to obtain the partial differential equations as shown in Eq (5).


$$\left\{\begin{array}{c} \frac{\partial Q}{\partial t}+gA\frac{\partial H}{\partial x}+\frac{f}{2DA}Q|Q|=0\\ C^{2}\frac{\partial Q}{\partial x}+gA\frac{\partial H}{\partial t}=0 \end{array}\right.$$ (5)

In Eq (5), *Q* denotes the flow rate in the pipeline; *A* expresses the cross-sectional area of the pipeline.


$$\left\{\begin{array}{c} L_{1}=\frac{\partial Q}{\partial t}+gA\frac{\partial H}{\partial x}+\frac{f}{2DF}Q|Q|=0\\ L_{2}=C^{2}\frac{\partial Q}{\partial x}+gA\frac{\partial H}{\partial t}=0 \end{array}\right.$$ (6)

At this point, after setting two, it multiplies *L*_2_ by the undetermined coefficient GGG, and then adds it to *L*_1_ to obtain *L*. The expression of *L* after sorting is shown in Eq (7).


$$L=(\frac{\partial Q}{\partial t}+\lambda C^{2}\frac{\partial Q}{\partial x})+\lambda A(\frac{\partial H}{\partial t}+\frac{1}{\lambda }\frac{\partial H}{\partial x})+\frac{f}{2DF}Q|Q|=0$$ (7)

If *H* = *H*(*x*,*t*) and *Q* = *Q*(*x*,*t*) are the solutions of the equation system shown in Eq (6), and the variable *x* is set as a function of time *t*, then the full derivative expressions of *Q* and *H* for *t* are shown in Eq (8).


$$\left\{\begin{array}{c} \frac{dH}{dt}=\frac{\partial H}{\partial x}\frac{dx}{dt}+\frac{\partial H}{\partial t}\\ \frac{dQ}{dt}=\frac{\partial H}{\partial x}\frac{dx}{dt}+\frac{\partial H}{\partial t} \end{array}\right.$$ (8)

At this point, if $\lambda C^{2}=\frac{dx}{dt}=\frac{1}{\lambda }$ is set, Eq (7) can be transformed into Eq (9).


$$\frac{dQ}{dt}=\lambda Ag\frac{dH}{dt}+\frac{f}{2DA}Q|Q|=0$$ (9)

In this way, the partial differential equation shown in Eq (7) can be converted into the ordinary differential equation shown in Eq (9) through $\lambda C^{2}=\frac{dx}{dt}=\frac{1}{\lambda }$. The expression shown in Eq (10) can be obtained through $\lambda C^{2}=\frac{dx}{dt}=\frac{1}{\lambda }$.


$$\frac{dx}{dt}=\pm C$$ (10)

Eq (10) indicates that if *x* and *t* are used as horizontal and vertical coordinates respectively; $\frac{dx}{dt}$ is denoted as two straight lines of $+\frac{1}{c}$ and $-\frac{1}{c}$ in the coordinate system, as shown in AP and BP in Fig 3. The characteristic lines of water hammer in the x-t coordinate system are shown in Fig 3. If Eq (10) is rewritten to the form shown in Eq (11).

dx=±C*dt (11)

**Fig 4:**
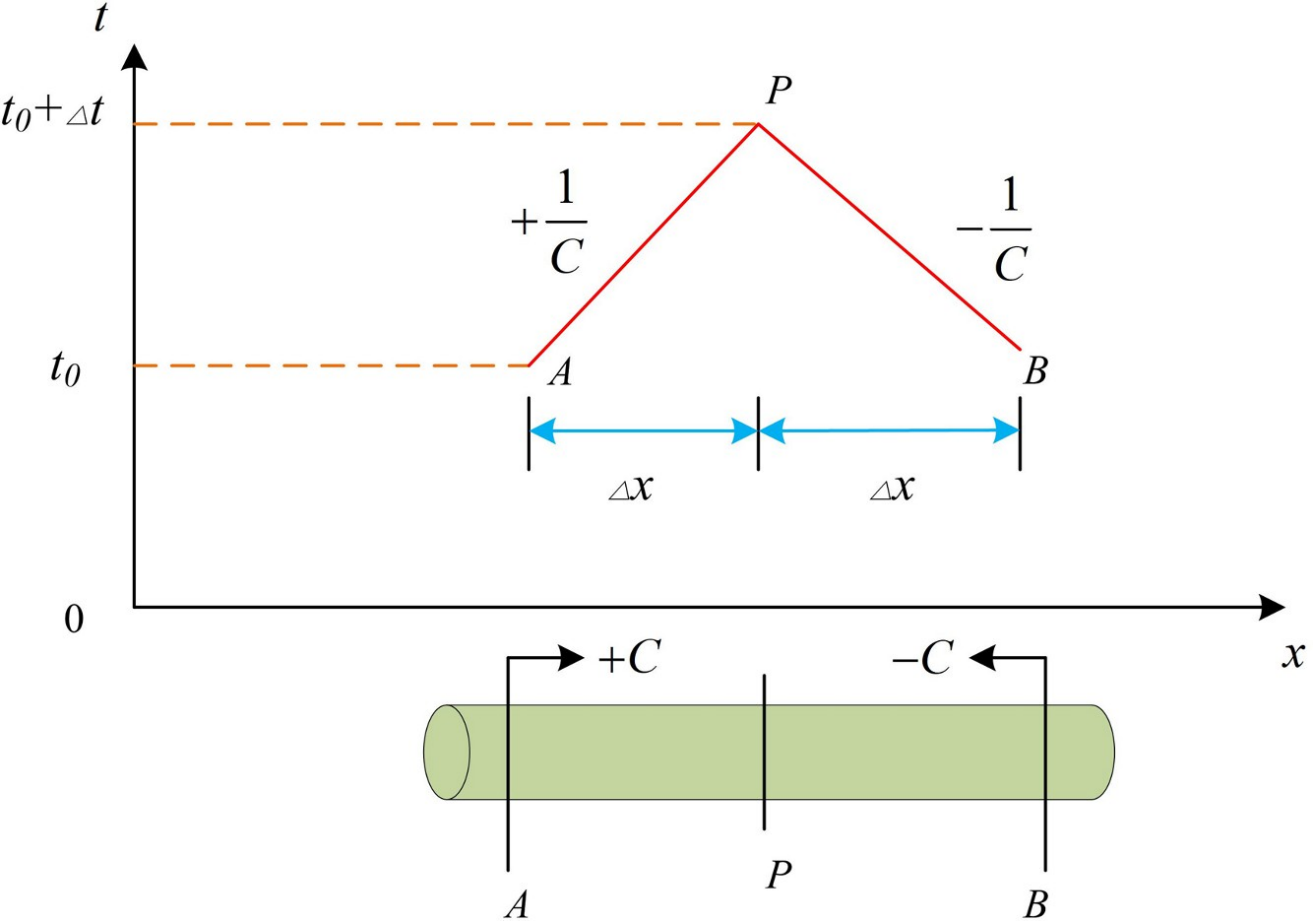
x-t Water hammer characteristic line in the coordinate system.

*dx* means the distance that the water hammer wave surface moves along the pipeline at wave velocity *C* during the *dt* period. In Fig 4, AP and BP indicate the positive and negative water hammer characteristic lines, respectively. By solving the water hammer characteristic lines, the relationship between flow rate in the pipeline and faucet can be determined, thus calculating the water hammer.

## 4. Water hammer protection measures based on MH pressure

Pressure regulating tower is considered the most effective and reliable means of hydraulic transient protection, which is essentially an open vertical shaft connecting pipelines to regulate hydraulic transient effects. During hydraulic transients, the pressure regulating tower can both buffer the water in the water buffer and inject water into the pipeline to avoid negative pressure in the pipeline. According to its basic structure, it can be divided into two types: single program and two-way. Among them, the single program pressure regulating tower is a water hammer protection measure based on the principle of MH pressure. This chapter focuses on the structure and protection principle of the one-way pressure regulating tower, to demonstrate the application of MH pressure in the hydraulic transition.

### 4.1 UPRT based on MH pressure

**Fig 5:**
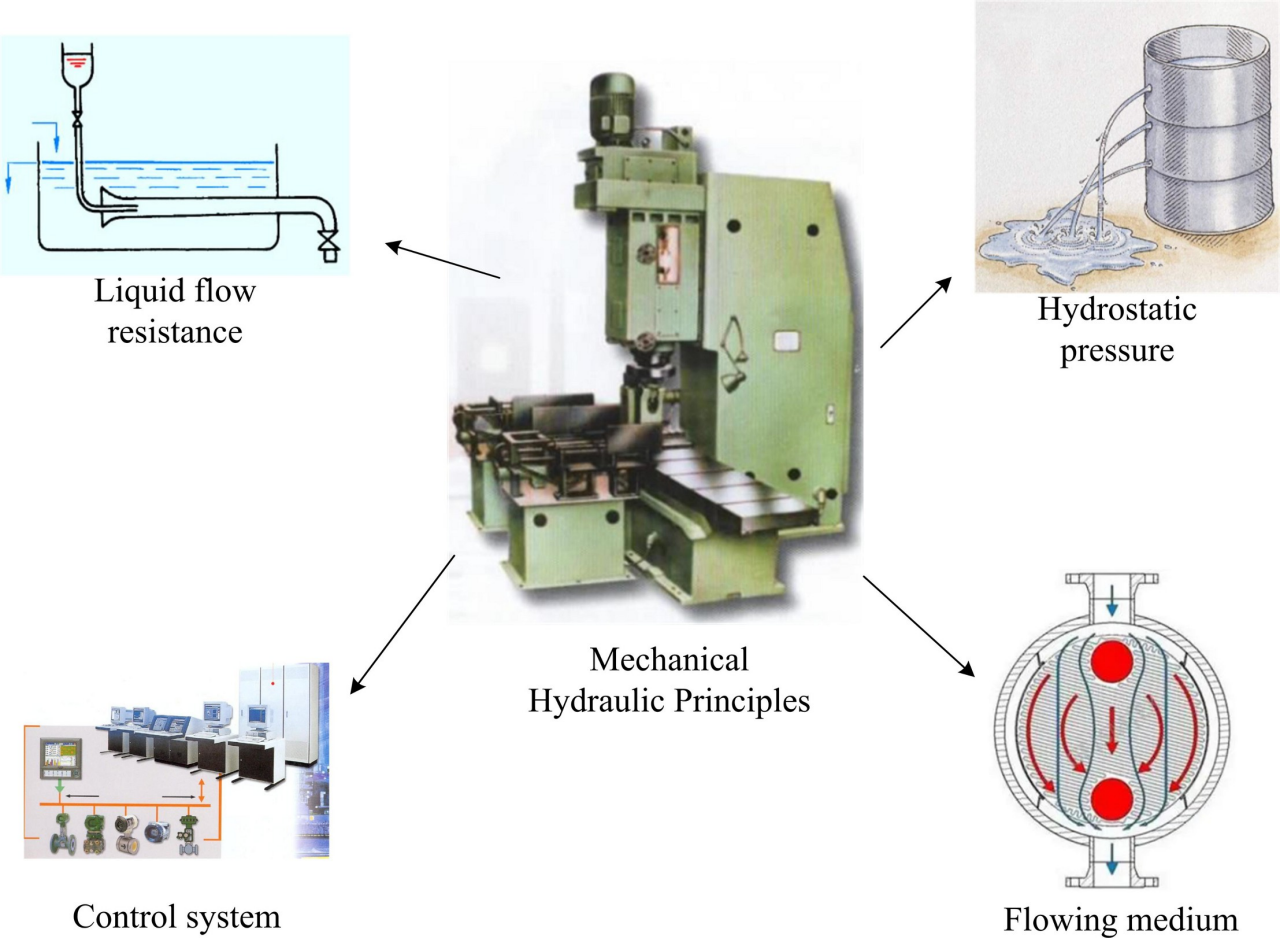
Main components of MH principle.

The principle of MH pressure refers to the use of liquid flow resistance and static pressure to control the pressure of mechanical equipment or systems. The main components of MH principles are liquid flow resistance, static pressure, flow medium, and control system. The main components of the MH principle are shown in Fig 5.

The liquid flow resistance in Fig 5 refers to the mechanical and physical resistance that affects the flow of liquid in equipment and systems, which can lead to a decrease in pressure. Static pressure refers to the influence of mechanical forces on the flow of liquid in equipment and systems, which can cause the liquid level to drop. Flowing medium refers to a liquid that can be composed of various media, such as air, oil, water, etc. In the principle of MH, oil is usually used as the flow medium. Because oil has a higher density than air and is prone to volatilization at high temperatures, oil is usually used in equipment and systems to maintain pressure and stability. The control system part refers to the MH principle that typically requires the use of control systems to control the pressure and flow of equipment and systems. These control systems can include components such as valves, pumps, controllers, etc. for precise control of equipment and systems. A UPRT is a hydraulic system based on MH principles, which regulates the pressure of equipment or systems by controlling changes in liquid pressure. In addition, a UPRT is also a device used to regulate the pressure of fluid conveying systems. When a fluid flows from one medium to another, it creates a pressure difference between the two flowing media, thereby maintaining a stable pressure in the fluid delivery system. In practical applications, UPRT based on MH can be used to control pressure fluctuations in fluid conveying systems, such as during the switching process of water pumps or fans. By changing the pressure of the fluid conveying system, the stability of the fluid conveying system during switching can be ensured. In addition, UPRT based on MH can also be used to control flow changes in fluid conveying systems, achieving flow regulation by adjusting hydraulic components and control systems. In summary, a UPRT based on MH principles is a water hammer protection device that regulates the pressure of the fluid conveying system by controlling the changes in liquid pressure to ensure the stability of the fluid conveying system during switching.

### 4.2 Research on water hammer protection mechanism of UPRT

**Fig 6:**
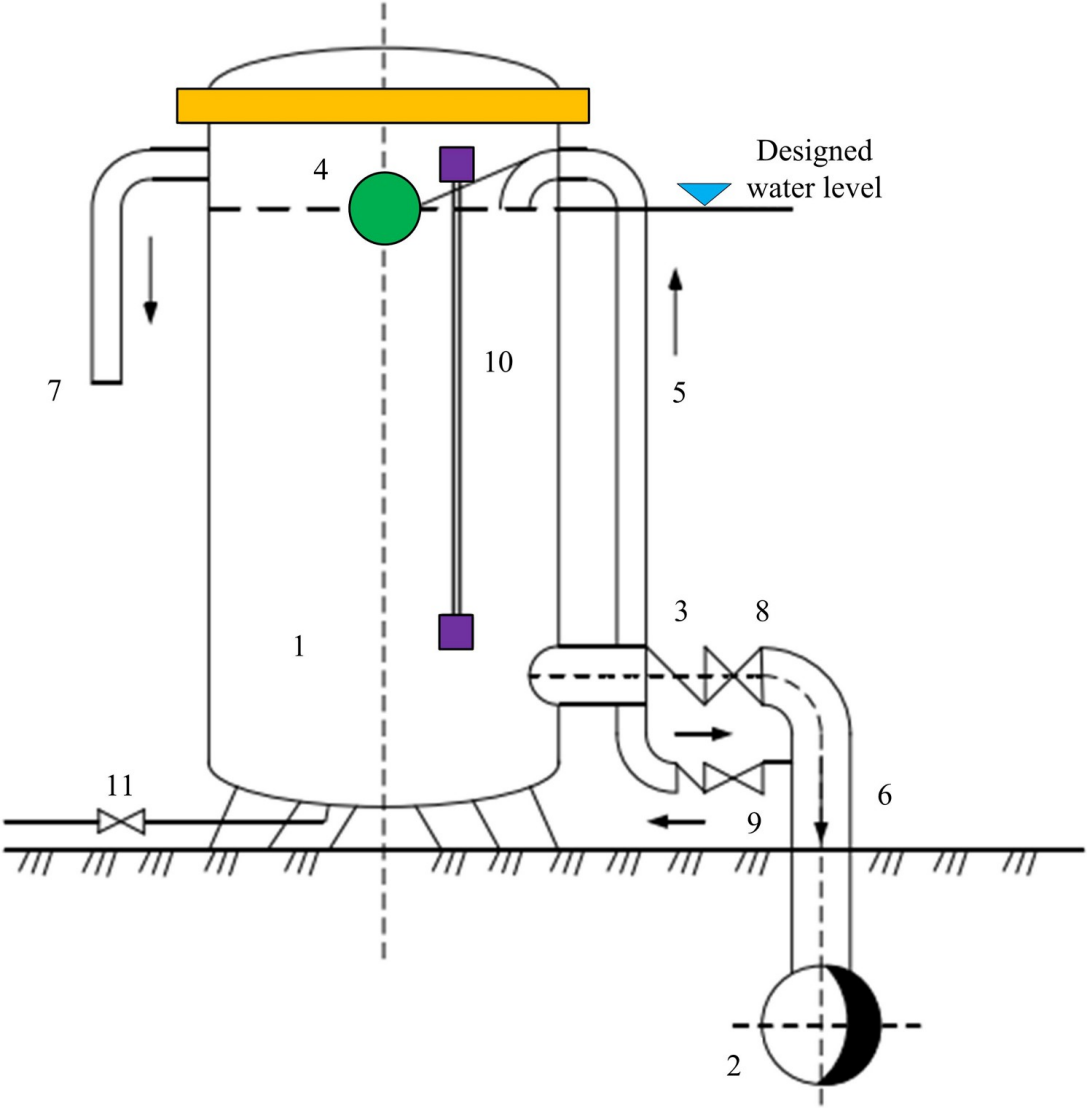
Specific structure of UPRT.

The working principle of a MH UPRT is to convert liquid pressure into piston motion through MH components, and adjust the pressure of the fluid conveying system by controlling the piston motion. The main components of an UPRT include hydraulic components, control systems, and auxiliary equipment. Hydraulic components are the core part of an UPRT, including pistons, oil cylinders, piston rods, etc. The control system is a system used to control the movement of hydraulic components and regulate the pressure of the fluid delivery system. Auxiliary equipment includes flow sensors, liquid level controllers, etc. The specific structure of the UPRT is shown in Fig 6.

**Fig 7:**
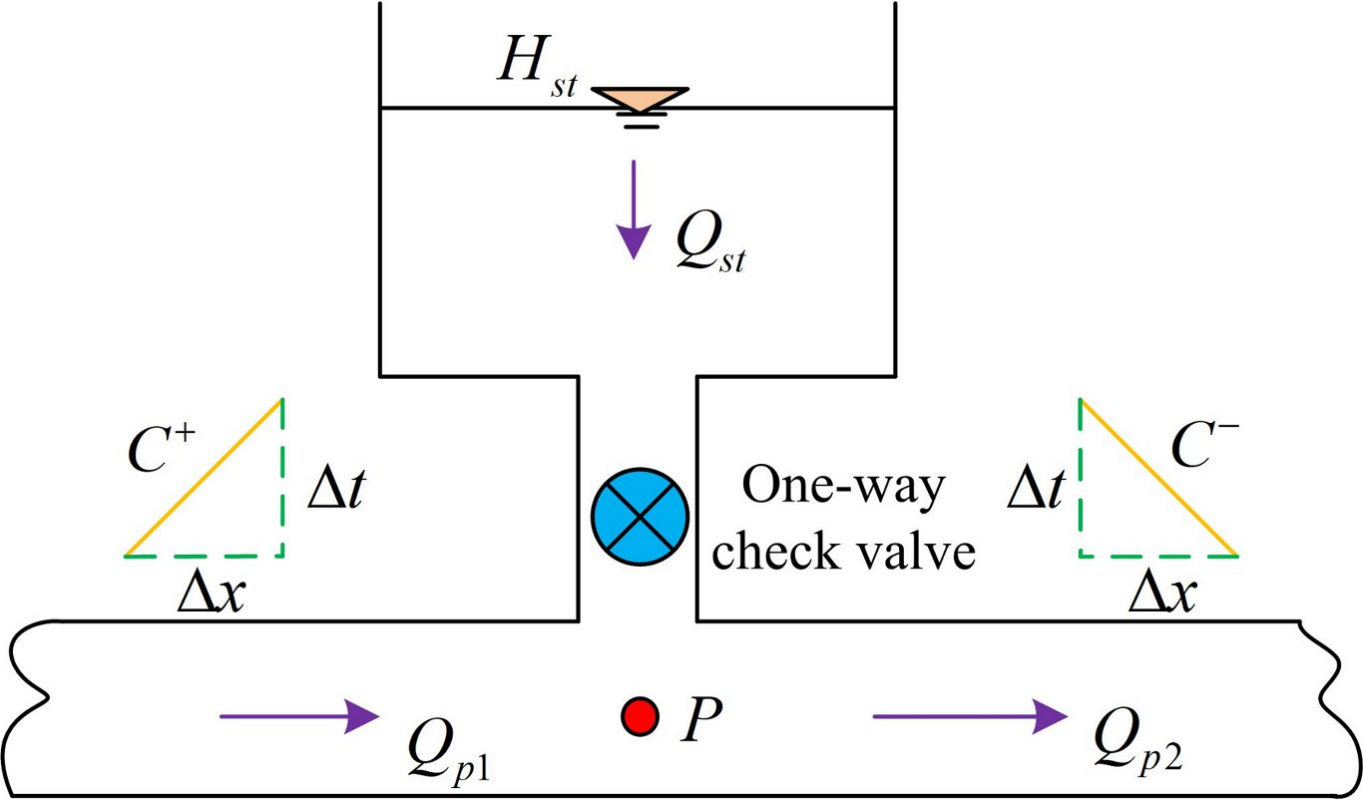
Mathematical model of UPRT.

**Fig 8:**
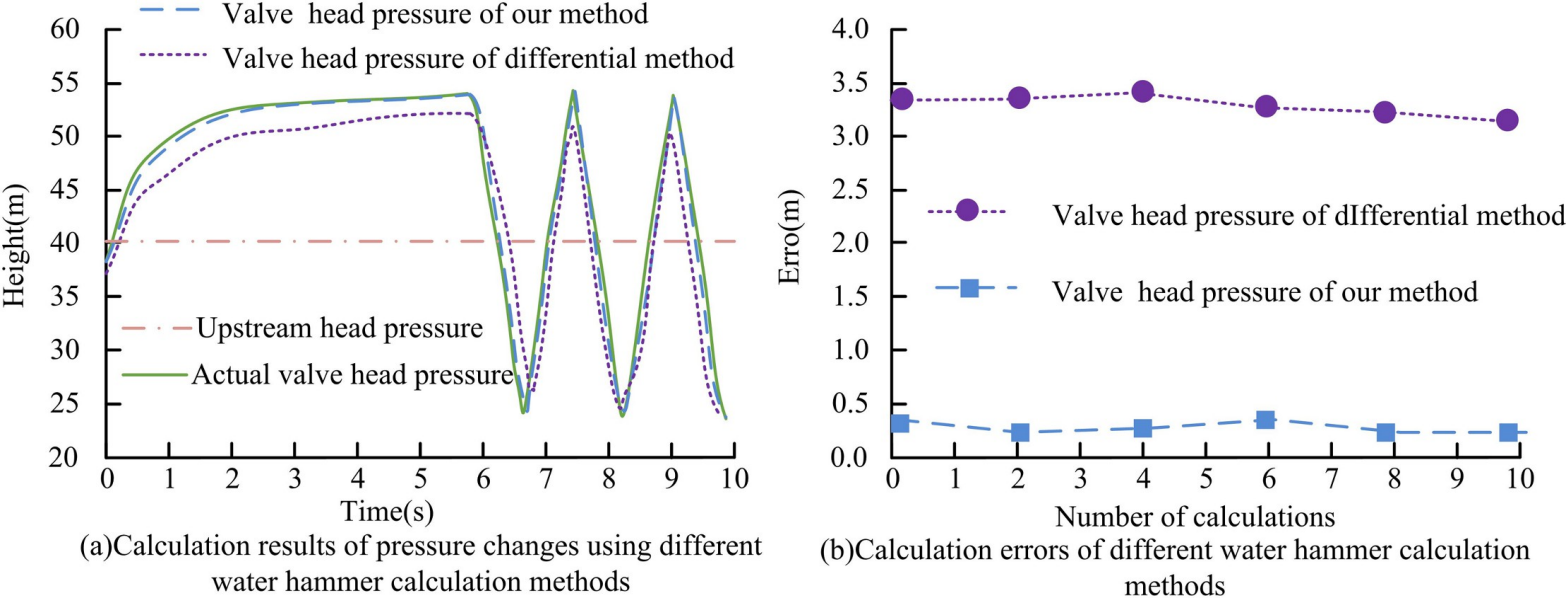
Validation results of water hammer calculation method effectiveness.

**Fig 9:**
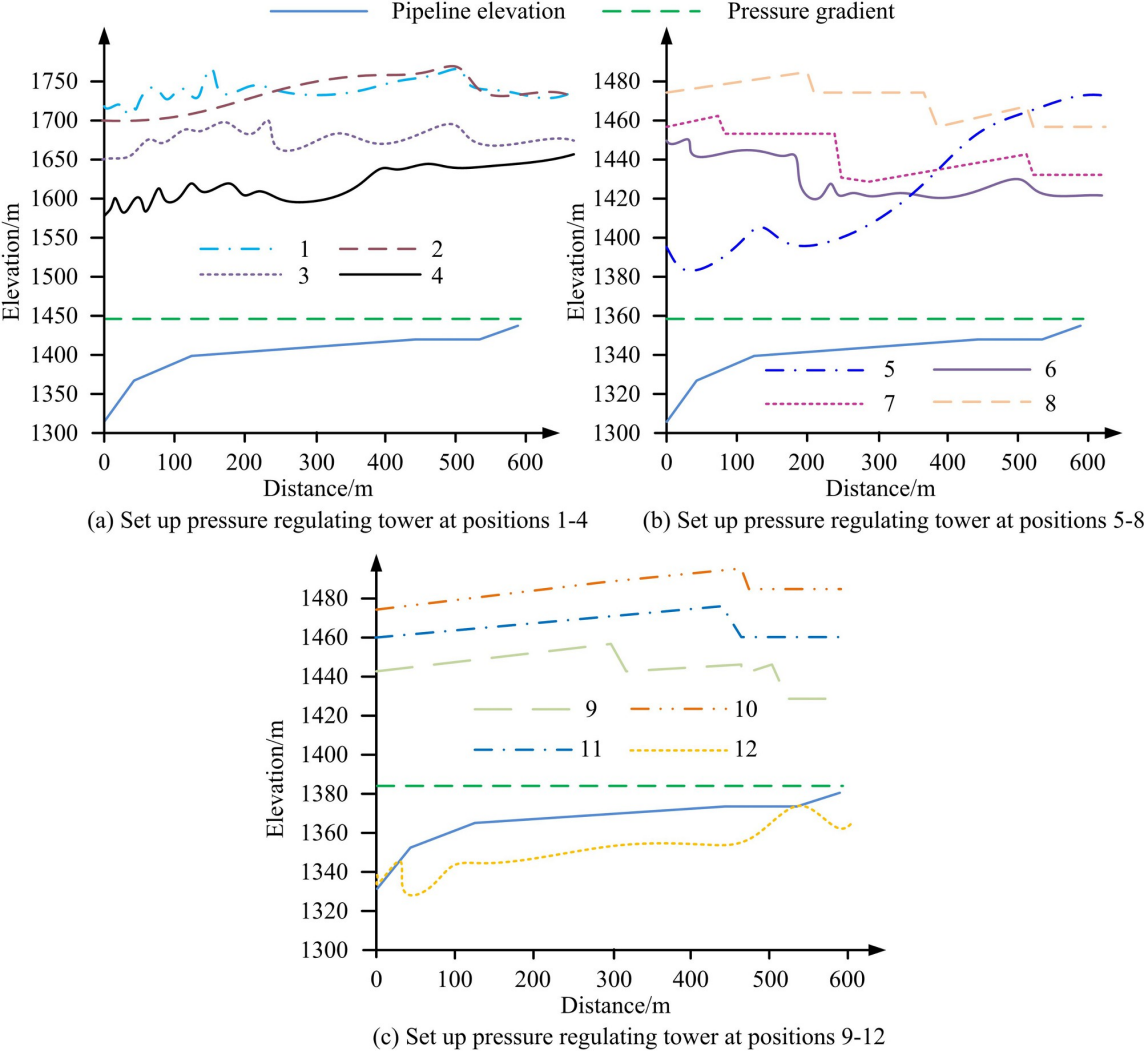
Simulation results of setting UPRT at different positions.

**Fig 10:**
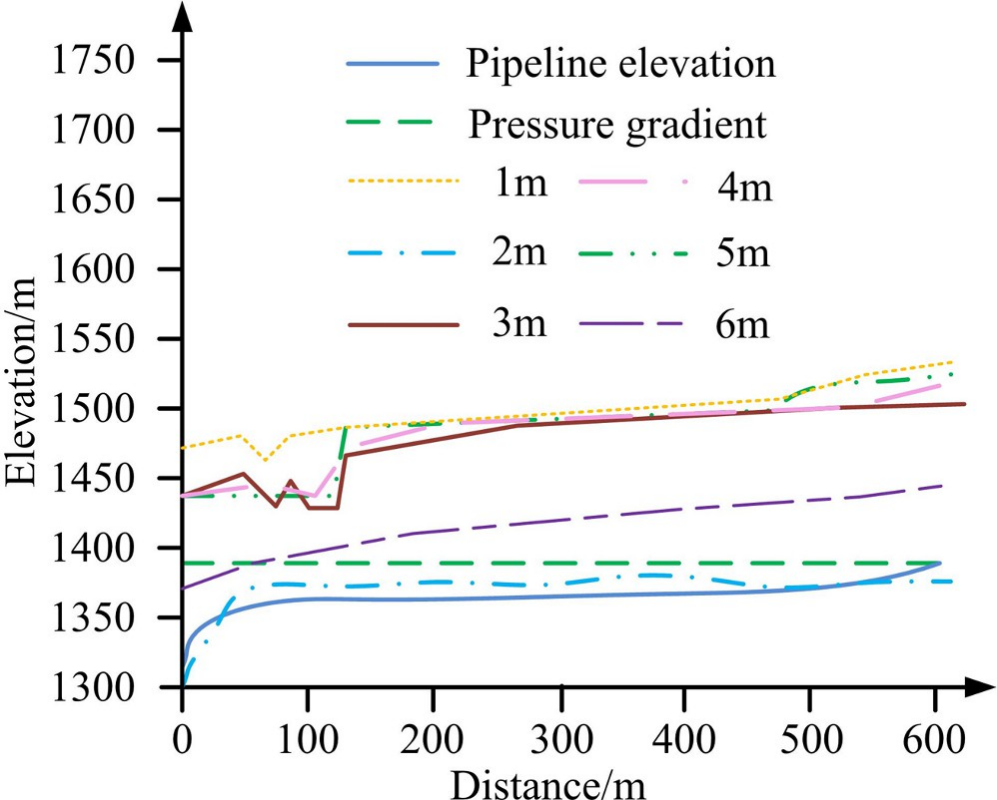
Simulation results of different volumes and initial water levels.

In Fig 6, 1, 2, 3, 4, 5, 6, 7, 8, 9, 10, 11 represent water tank, main pipe; check valve, floating ball, inlet pipe, water injection pipe, overflow pipe, gate valve, check valve of the inlet pipe; 10 represents, water level gauge and emptying tube, respectively. An UPRT is a buffer type water hammer protection device, whose main function is to avoid the separation of negative pressure water columns in the pressure pipeline. When the pressure inside the pipeline decreases, the pressure regulating tower will quickly replenish water, thereby avoiding or reducing negative pressure in the pipeline and reducing pressure rise. Therefore, the pressure regulating tower should be set at the position where negative pressure is most likely to occur, and the closer it is to the pump edge, the better. But when the pressure difference of the pipeline is large, the height of the pressure regulating tower will also increase, which will increase the engineering cost. So when choosing, it is important to compare from an economic perspective. Normally, a surge tower includes a tower water chamber, auxiliary branch pipes, and valve components. The opening and closing of the directional valve are achieved through the main pipe pressure, and the directional valve is usually closed.

During the hydraulic transition period, if the water head in the regulating tower pipe is lower than the water level under the regulating pressure, the reverse valve will open, and the water in the tower will enter the main pipe, so that there will be no excessive negative pressure or water column detachment in the main pipe. When the pressure in the main pipe gradually exceeds the water level in the tower, the backflow valve will quickly close or slowly close. In the case of a slow flow valve, the water level in the pressure regulating tower is maintained through a branch pipeline in the main pipe, and after the water level in the container reaches normal level, the outlet of the water supply branch pipeline is closed through a float valve. The design calculation of the pressure regulating tower requires that the pressure regulating tower has sufficient capacity to supply the required makeup water to prevent negative pressure in the main pipe from causing water column separation. The minimum water level of the pressure regulating tower should have sufficient pressure to replenish water to the main pipe, and the maximum water level should have sufficient water replenishment, while also ensuring that the structure of the pressure regulating tower is economically reasonable. The water supply short pipe of the pressure regulating tower can ensure its water supply speed by relying on the gravity of the water body during the water supply process. The installation position, capacity, water level height, size of water injection pipe and size of waterproof hammer of UPRT shall be simulated by computer, compared and finally determined.

### 4.3 Mathematical model construction of UPRT

Based on the structure and operating mechanism of the UPRT mentioned above, a mathematical model of the UPRT is constructed, as shown in Fig 7.

As shown in Fig 7, if the boundary node numbers of the inlet and outlet water pipes are 1 and 2 respectively, the relationship between the water level and flow rate of the UPRT is shown in Eq (12).


$$\frac{dH_{st}}{dt}=\frac{Q_{st}}{A_{st}}$$ (12)

In Eq (12), *H*_*st*_ and *A*_*st*_ respectively denote the water level and cross-sectional area of the one-way surge tank; *Q*_*st*_ indicate the instantaneous flow rate from the UPRT. By integrating both sides of Eqs (12) and (13) is obtained.


$$H_{st}=H_{s0}+\frac{\left(Q_{st}+Q_{s0}\right)}{2}\frac{\Delta t}{A_{st}}$$ (13)

In Eq (13), Δ*t* means the time step size. When considering the local head loss at the one-way check valve, the head balance equation and flow continuity equation of the UPRT are shown in Eq (14).


$$\left\{\begin{array}{c} H_{P}=H_{st}-kQ_{st}|Q_{st}|\\ Q_{p2}=Q_{st}+Q_{p1} \end{array}\right.$$ (14)

In Eq (14), *k* and *H*_*P*_ respectively refer to the hydraulic loss coefficient of the one-way check valve and the transient head at the bottom of the UPRT; *Q*_*p*1_ represents the transient flow into the node; *Q*_*p*2_ expresses the transient flow at the outflow node. Among them, the pipeline compatibility equation is shown in Eq (15).


$$\left\{\begin{array}{c} H_{P}=C_{P1}-BQ_{P1}\\ H_{P}=C_{M2}-BQ_{P2} \end{array}\right.$$ (15)

By combining Eqs (13), (14) and (15), the solution of *Q*_*st*_ can be obtained as shown in Eq (16).


$$Q_{st}=\frac{(2C_{2}-B)-\sqrt{{\left(2C_{2}-B\right)}^{2}-8k(C_{3}-2C_{1})}}{4k}$$ (16)

In Eq (16), *C*_1_ = *H*_*s*0_ + *Q*_*s*0_Δ*t* / (2*A*_*st*_), *C*_2_ = Δ*t* / (2*A*_*st*_), *C*_3_ = *C*_*P*1_+*C*_*M*2_. It substitutes the instantaneous flow rate *Q*_*st*_ obtained from the UPRT into Eqs (13), (14), (15), and (16) can obtain *H*_*P*_, *H*_*s*_, *Q*_1_, and *Q*_2_. After determining the various data of the UPRT using the above mathematical model, it can be applied to reduce the WHE in the water supply pump station.

## 5. Numerical simulation of the HTP of the pumping station in the Yumenkou East Expansion Project

To better analyze the application effect of UPRT based on MH pressure on the HTP of water supply pump engineering, a simulation system and water hammer model was constructed in this chapter, and the water hammer calculation method would be verified. In addition, the parameters of the individual surge tank were optimized to compare and study the practical application effects of this water hammer protection measure.

### 5.1 Construction of simulation system and verification of water hammer calculation method

To analyze the actual effect of UPRT on the water supply pump station, a numerical simulation calculation software for the HTP of the Yumenkou East Expansion Project was developed based on the situation of the Yumenkou East Expansion Project and the principle of HTP in the water supply project. The specific parameters of the software system are shown in Table 2.

**Table 2:** The experimental basic environmental parameters.

Parameter variables	Parameter selection
Operating system	Windows 7
Programming Language	Visual Basic 6.0
Database Management System	Microsoft SQL Server 2000

As shown in Table 2, the operating platform of the software system researched and developed was Windows 7, the programming language was Visual Basic 6.0 software, and the Database management system used Microsoft SQL Server 2000. The software was used to simulate the boundary adjustment at the pump and valve, and calculate the maximum backflow, maximum and minimum water hammer pressure, to establish the mathematical model and hydraulics model for water hammer calculation of complex pump system, and then complete the simulation of pump station water hammer. To verify the effectiveness of the characteristic line water hammer calculation method proposed in the study, the study took the calculation example in the third edition of hydraulics textbook of Tsinghua University as the experimental object, and took the actual pressure curve error as the index to carry out comparative experiments on the characteristic line water hammer calculation method proposed in the study. The comparison method was the water hammer calculation method based on the difference method. It was known that a horizontal pipeline had a diameter of 4.6m, a length of 316m, a wall thickness of 0.02m, a head loss coefficient of 0.025, and a wave velocity of 790m/s. Initially, the pump station valve was fully open, with a maximum flow rate of 40m^3^/s and a valve closure time of 6s, which was a linear closure. The internal water hammer pressure was calculated for 10s. The calculation results are shown in Fig 8.

Fig 8(A) shows the valve water hammer pressure calculation results of different water hammer pressure calculation methods. Its principle is to calculate the rate of change of the flow rate on the basis of determining the speed and time when the valve is closed, that is, the slope of the flow rate, and finally determine the water hammer pressure. From Fig 8(A), the water hammer pressure head at the initial valve was 38.8m, and the maximum water hammer pressure head was 54.06m. The water hammer pressure curve and actual water hammer pressure curve obtained by the method proposed in the study, and the water hammer pressure of the difference method, the maximum water hammer pressure head was 54.02m, which was well matched with the actual results. Fig 8(B) shows the calculation error of valve water hammer pressure for different water hammer pressure calculation methods. From Fig 8(B), the calculation error of the method proposed in the study was 0.36m, and the calculation error of the difference method was 3.02m lower. It can be seen from the above results that the water hammer method proposed in the study is more accurate than the traditional difference method in considering the flow variation when the valve is closed. This may be because the characteristic line method more fully considers the details of the flow change, by calculating the slope of the flow rate to more accurately estimate the change in water hammer pressure. This method can obtain a water hammer pressure curve which is in good agreement with the actual results. In contrast, the traditional difference method may not fully consider the details of the flow change, resulting in a large error between the calculated results and the actual results. Through the improvement of characteristic line method, more accurate calculation results of water hammer pressure can be obtained, and the evaluation accuracy of water hammer pressure of valve can be improved. Based on the above results, the proposed method could effectively calculate water hammer pressure, and its computational performance was superior to traditional differential water hammer calculation methods.

### 5.2 Analysis of the layout conditions of the UPRT position

To analyze the specific layout position of the UPRT, the research and design software was used to simulate the working conditions at different positions of the surge tank, quantify the protection effect of positive and negative pressure water hammer on the pipeline, and then compare and select the optimal position. Based on the pump station of the Yumenkou Water Lifting East Expansion Project, simulation analysis was conducted on pipelines at different locations. An UPRT with a diameter of 4m, a makeup pipe with a diameter of 450mm, and an initial water level of 6m were set up to simulate the pump stopping water hammer. The simulation results are shown in Table 2, and 1–12 in the figure represent 12 different locations.

From Fig 9, when UPRTs were installed at different positions, the maximum pressure value of the pipeline fluctuated significantly. Among them, when UPRTs were installed at 5 positions, the positive pressure amplitude of the pipeline changed the most, from 1396m to 1476m. When setting pressure regulating towers near positions 1, 2, 10, 11, and 12, the maximum pressure and air pocket volume along the pipeline were equivalent to those without protective measures, so setting pressure regulating towers at these positions could not reduce the WHE. To further select suitable locations, the study also conducted statistical analysis on the water hammer calculation results of UPRTs deployed at different locations, as shown in Table 3.

**Table 3:** The experimental basic environmental parameters.

Serial Number	Position	Maximum pressure of pipeline(m)	Minimum pressure of pipeline(m)	Maximum steam capacity(m^3^)
1	32+736	223.26	-9.68	13.415
2	33+126	215.38	-9.68	13.337
3	33+458	184.56	-9.68	7.667
4	33+769	148.47	-9.68	5.698
5	34+089	103.24	-9.68	4.012
6	34+421	158.68	-9.68	5.023
7	34+833	166.46	-9.68	5.679
8	35+084	185.77	-9.68	8.326
9	35+381	203.47	-9.68	11.176
10	35+715	213.54	-9.68	12.659
11	36+056	218.32	-9.68	13.358
12	36+368	191.56	-9.68	14.128

From Table 3, when a UPRT was installed at position 5, the highest positive pressure value of the pipeline was 103.13m, which was much smaller than the highest positive pressure value of the pipeline with a UPRT installed at other positions. In addition, the maximum steam volume when setting up a UPRT at 5 locations was also the smallest, with a value of 4.012m^3^, which significantly damaged the pressure generated by the bridging water hammer. This was consistent with the actual location of the UPRT in the project. Therefore, location 5 could be selected as the location for setting up the unit pressure regulating tower in this study.

### 5.3 Optimization analysis of parameters for UPRT

To better utilize the UPRT to control the pipeline water hammer pressure of water supply pump engineering, research needed to first optimize the parameters of the UPRT. This study aimed to optimize the three parameters of volume, initial water level, and makeup pipe diameter in a UPRT. Simulation software designed through research was used to compare the water hammer pressure under different initial water levels and makeup pipe diameters, to find the optimal parameter values. The simulation results of different initial water levels in the simulation software are shown in Fig 10.

**Fig 11:**
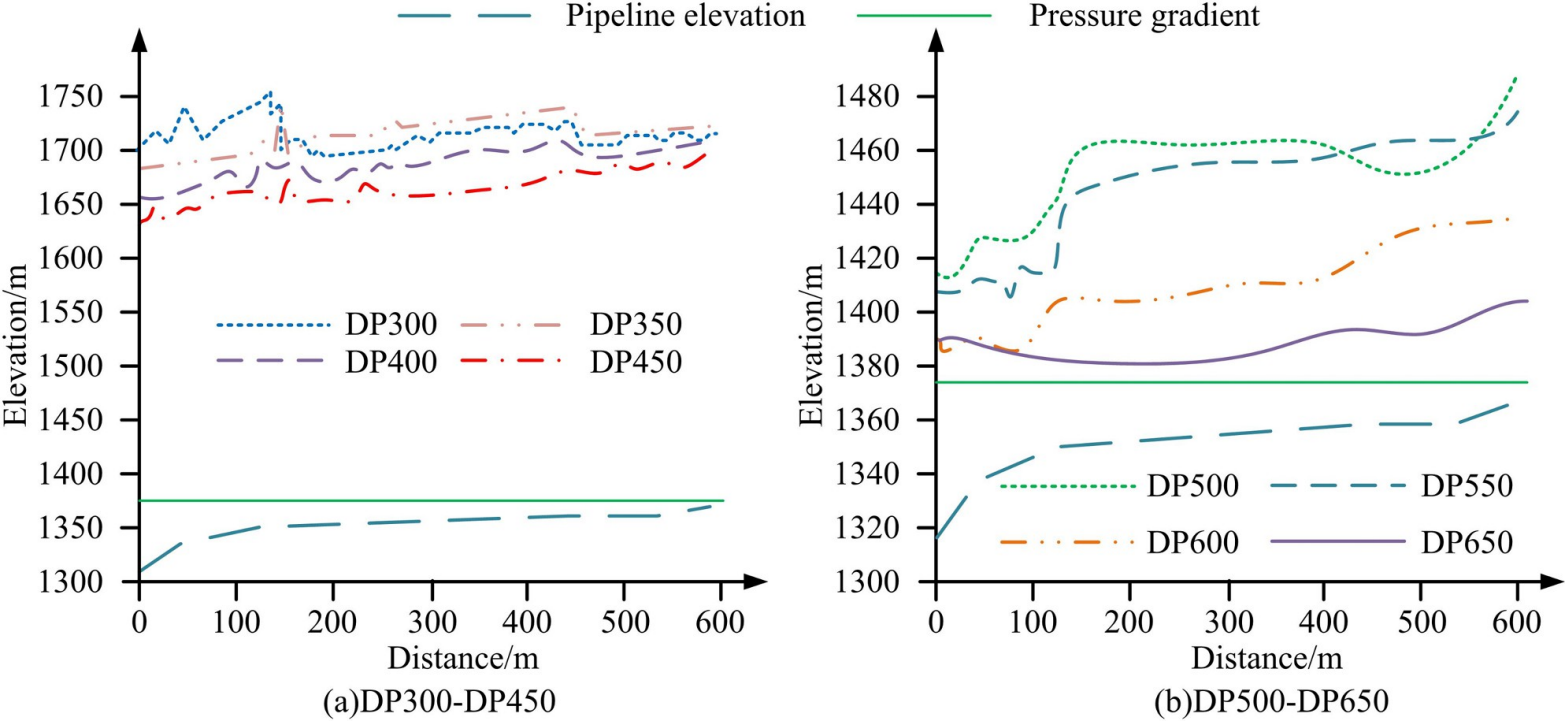
Simulation results of simulation software for different water supply pipe diameters.

From Fig 10, under different initial water levels inside the tower during an accident, the maximum and minimum pressure values along the pipeline decreased accordingly. However, when the initial water level inside the pressure regulating tower was 2m, the pressure value of the pipeline was significantly lower than the water hammer pressure at other water levels. Therefore, the initial water level of 2m was selected for this experiment. The simulation results of different water supply pipe diameters using simulation software are shown in Fig 11.

From Fig 11, as the diameter of the make-up water pipe in the surge tank increased, the maximum positive water hammer pressure gradually decreased. When the diameter of the make-up water pipe was below DN600, the maximum pressure envelope line of the pipeline almost overlaped with the steady-state pressure line. Therefore, using a DN600 water supply pipe diameter, there was no cavity inside the pipe and the control effect of the maximum pressure of the pipeline is better. In conclusion, using an UPRT with an initial water level of 2m and a makeup pipe diameter of 600 mm could achieve good water hammer protection effect.

### 5.4 Analysis of protective effects under different protective measures

**Fig 12:**
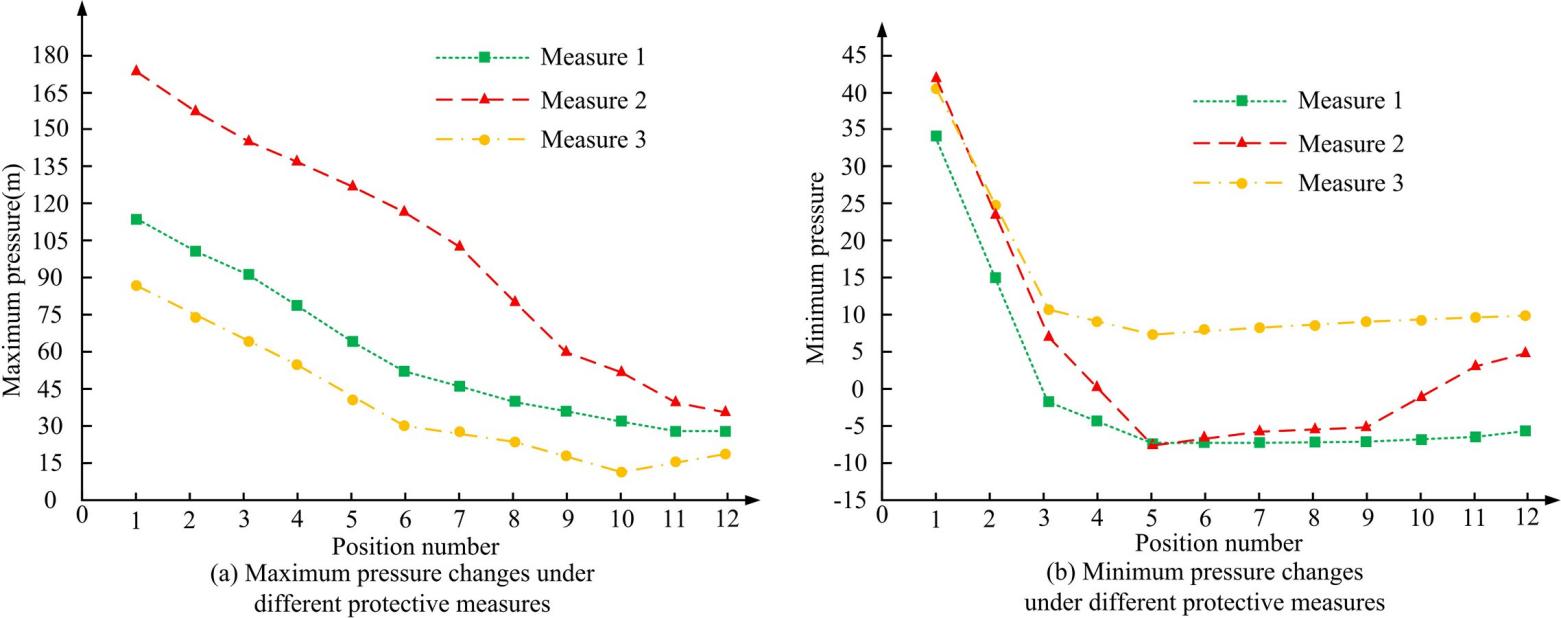
Pressure changes at different positions under three different protective measures.

To analyze the protective effect of the proposed water hammer protection measures based on UPRT (measure 1), the study compared their protective effects with traditional protective measures (measure 2) and water hammer protection measures based on vacuum breaking valves (measure 3). The study used simulation software to compare the pressure changes at each position under the three different protective measures mentioned above, to analyze the protective effect of each protective measure. The pressure changes at each position under the three different protective measures are shown in Fig 12.

**Fig 13:**
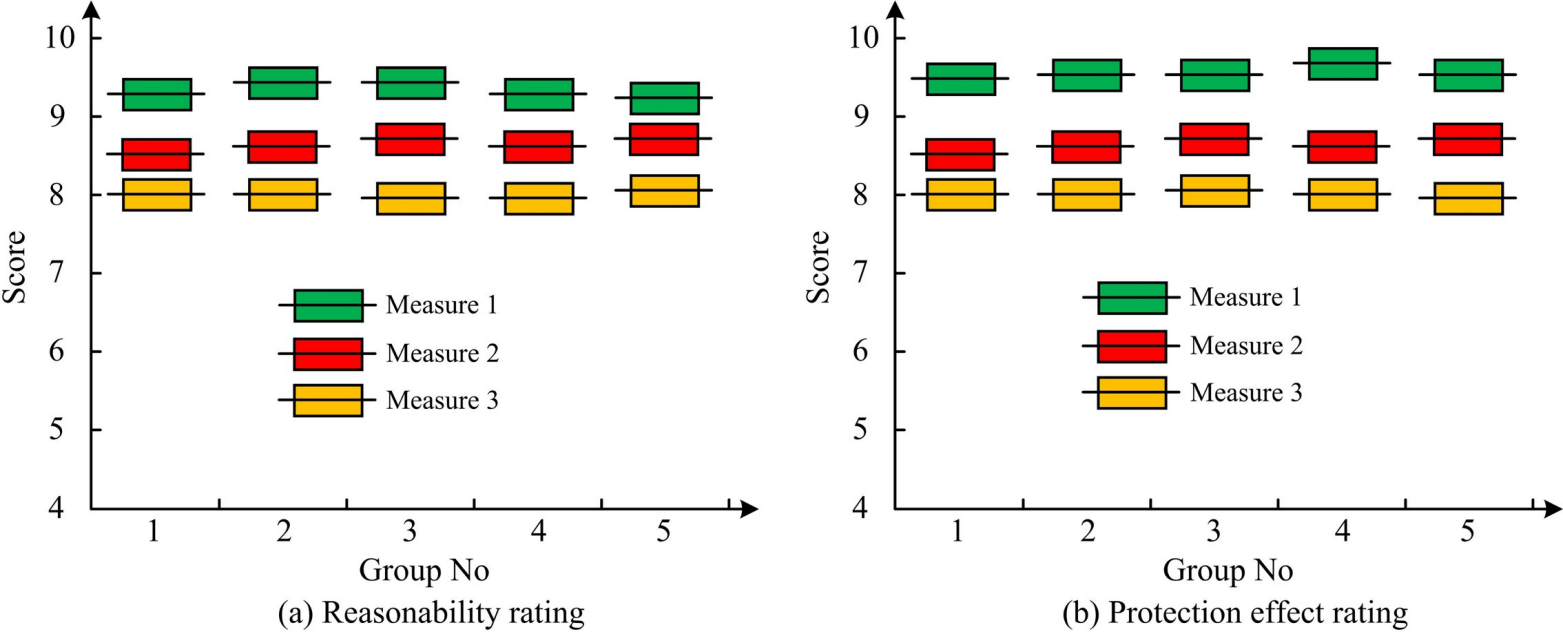
Scoring results of the protective effects of three types of protective measures by relevant technical personnel.

According to the simulation results in Fig 12(A), as the position number increased, the maximum pressure of the water hammer under all three protective measures decreased, and protective measure 1 had the most significant reduction in the maximum water hammer pressure of the pipeline. Its maximum positive pressure at position 10 was the lowest, at 14.8m, which was much lower than 31.3m of protective measure 2 and 63.8m of protective measure 3. From the simulation results in Fig 12(B), different protective measures had different control effects on negative pressure. Among them, measures 2 and 3 had poor control effects on negative pressure at different positions of the water supply pump, while the minimum pressure at different positions of protective measure 1 was mostly within the range of 5-15m. In summary, based on the actual water supply pump project, the water hammer protection effect of protective measure 1 was better. To further analyze the effectiveness of the three protective measures, relevant technical personnel were selected to score the effectiveness of the three protective measures, and their application effects were analyzed based on the scoring results. The evaluation results of the protective effects of the three protective measures by relevant technical personnel are shown in Fig 13.

From Fig 13(A), the rationality scores of protective measure 1 by five groups of relevant technical personnel were all above 9 points, and their average rationality score was 9.2 points, which was higher than 8.6 points of protective measure 2 and 7.9 points of protective measure 3. From Fig 13(B), the protective effect scores of the five groups of relevant technical personnel on protective measure 1 were all above 9 points, and their average protective effect score was 9.4 points, which was higher than 8.7 points of protective measure 2 and 8.0 points of protective measure 3. The above results indicated that relevant technical personnel believed that the protective effect of protective measure 1 was better than that of protective measures 2 and 3. In summary, the proposed water hammer protection measures based on UPRTs could effectively protect the WHE of water supply pumps, and could be applied in practice to reduce the occurrence rate of water hammer in water supply pump engineering.

## 6. Conclusion

This research was based on the simulation analysis of HTP of MHs. Through the simulation and analysis of the water hammer pressure during the valve closing process, simulation software was designed according to the actual situation and relevant principles, and the application effect of the unidirectional pressure regulator in the specific project of the water supply pump was analyzed by using this software. So a better way was chosen to control the water hammer effect (WHE) in the water supply pump project. The simulation experiment proved that the following conclusions were drawn: 1. The water hammer pressure was caused by the conversion of fluid kinetic energy into pressure energy when the valve was closed, and its magnitude was related to the closing speed of the valve, the characteristic parameters of the pipeline and the properties of the fluid, etc. Under this protective measure, the maximum positive pressure at position No. 10 was the lowest, which was 14.8m. 2. In the process of closing the valve, the water hammer pressure rose rapidly in the initial stage, then the pressure gradually decayed, and finally reached a stable state. The calculation error of the characteristic line method was 0.36m, which was superior to the traditional method. 3. The closing speed of the valve had a great influence on the water hammer pressure. The faster the closing speed, the greater the water hammer pressure. 4. The characteristic parameters of the pipeline, such as length, diameter, material, etc., would also affect the water hammer pressure. Longer pipes or smaller diameters would increase the amount of water hammer pressure. In summary, the HTP simulation analysis research based on MHs has carried out an in-depth analysis of the water hammer pressure during the valve closing process, providing a theoretical basis for valve design and working parameter selection in practical engineering. In practical applications, the valve closing speed should be reasonably controlled according to the specific situation, and the influence of pipeline characteristics and fluid properties should be considered to reduce the damage of water hammer pressure on pipelines and equipment.
